# The Treatment of Pneumonia

**Published:** 1894-03-24

**Authors:** T. R. Bradshaw

**Affiliations:** Assistant Physician to the Liverpool Royal Infirmary


### MEDICAL PROGRESS AND HOSPITAL CLINICS.

[*The Editor will be glad to receive offers of co-operation and contributions from members of the profession. All letters should be addressed to* THE EDITOR, THE LODGE, PORCHESTER SQUARE, LONDON, W.]

### THE TREATMENT OF PNEUMONIA.

By T. R. BRADSHAW, B.A., M.D., M.R.C.P., Assistant Physician to the Liverpool Royal Infirmary.

Pneumonia is a term that is somewhat loosely employed to include a variety of pathological states of the lung, all of which, however, possess this feature in common, that they are attended by an increase of the solid or fluid constituents of the organ, and by a corresponding diminution of the air spaces. These various conditions are usually distinguished by adjectives describing either the character of the consolidation or some other clinical or pathological peculiarity. Many of them arise in association with other and more obvious disorders; thus, catarrhal pneumonia arises in association with bronchitis, caseating pneumonia with phthisis, and "hypostatic" pneumonia in the course of asthenic fevers. The treatment of these forms of pneumonia merges into that of the disorders with which they are associated, and will not be specially dealt with here. The remarks that follow refer to pneumonia which has come on independently of any other obvious disease, and which is, in the words of Dr. Fagge, "idiopathic in its origin, acute in its course, lobar in its extent, basal in its usual distribution, and fibrinous in the character of its exudation."

Whether we look upon pneumonia as a local inflammation in the lung, attended with symptomatic fever, or as a general infective disorder due to the presence of a specific poison, and accompanied by a local lesion, we have to admit that our treatment must be mainly directed to the constitutional state. It is doubtful how far, if at all, the extent of consolidation is influenced by any means we can adopt, and it is generally admitted that the so-called antiphlogistic measures that were formerly adopted with the view of controlling the local inflammation were not only useless, but often disastrous in their results. Without going so far as to say that the line of treatment to be adopted in a given case is independent of the local condition of the lungs, we must admit that the indications derived from this source are mostly of only secondary importance.

It follows from these considerations that before we can arrive at a rational line of treatment for pneumonia we must learn what is the constitutional state in the disease, and this will be determined in any given case by the antecedent state of the patient's health, and by the constitutional effects of the poison in the blood or of the local lesion, or of both combined. What, then, is commonly the state of health of those who become the subjects of pneumonia? Leaving out of account the subjects of chronic heart and kidney disease, and the aged, it will be found that, at least in hospital practice, the great majority of those who are admitted with pneumonia have been in a low state of health for some time before the occurrence of the rigor that marked the invasion of the acute disease. Many have been the subjects of chronic alcoholism, privation, or overwork, and many give a history of cough and expectoration for some weeks. Antecedents such as these point to a constitutional state which needs support rather than depletion.

Turning to the constitutional effects that are directly dependent on the presence of the disease itself, we may broadly divide them into early and late. At the outset we have usually marked febrile reaction, a sudden rise of temperature, flushed cheeks, pungent skin, headache, and distress. The temperature commonly reaches 104 deg., but without much exceeding it, the pulse is bounding and about 100, and there is usually no delirium. In favourable cases this condition may go on until the crisis, but in others it is succeeded or may be replaced from the first by a state of which the prominent feature is prostration. The temperature may or may not be as high as before, but the pulse is quicker and weaker and the heart sounds are obscure, the trunk muscles are unequal to the calls arising from the embarrassed respiration, pallor or cyanosis appears, and clammy perspiration breaks out.

Now, we shall not be far from the truth if we say that, as a rule, the more marked the stage of febrile reaction the more hopeful is the prognosis in the majority of cases; that, unlike typhoid and acute rheumatism, the fever of pneumonia is not in itself an element of danger, and that the chief ground for alarm is the appearance of prostration either early or to a great degree. We may conclude from what has been said that the maintenance of the patient's strength until the arrival of the natural termination of the disease is the main end to be kept in view in the treatment of pneumonia, and that the state of the heart muscle especially calls for support. Further, we may have to treat symptoms as they arise, to relieve pain, procure sleep, regulate the bowels, and moderate excessive feverishness.

Having thus reviewed the principles that form a rational basis for the treatment of this disease, I will describe the means I generally employ to carry them out.

The patient is as quickly as possible put to bed, the usual bath being dispensed with, and any washing that is needed being done afterwards. If the breathing is much embarrassed he is allowed to be propped up in bed. Food is given in small quantities every two hours, the staple food being milk, sometimes supplemented by beef-tea or eggs. As to the value of beef-tea as a food I will say nothing here, further than to observe that while I never trust to it to the exclusion of milk, I think that, as Dr. Lauder Brunton has suggested, it may contain substances that are useful stimulants to the heart. The food must be given regularly by night as well as by day, and cooling lemonade, "imperial," or water may be taken in moderation. There is no objection to a cup of tea in the morning, or to the sucking of a few grapes or an orange, only it is difficult to get hospital patients to reject the pulp of the latter. When the skin is harsh and burning, as it generally is, tepid sponging is resorted to once or twice in the day.

As regards local applications to the chest, most cases are poulticed during some part of their course. This is, however, omitted in the case of old, weakly people, who would be likely to receive more harm from the disturbance than good from the application; in fact, the curative influence of poultices in this disease is more than doubtful, except for the relief of pain in the side. When the latter is absent it is generally enough to wrap up the chest in a layer of wadding. The use of alcoholic stimulants is one of the points in treatment about which a great deal may be said. The way in which alcohol acts in disease we probably do not altogether understand. The view put forth by Dr. Coupland—namely, that it induces a state of partial narcotism, with a certain degree of oblivion to his distresses in the mind of the patient, perhaps contains an important element of truth. At any rate, almost all cases of this disease receive a certain amount of alcohol, and in many its beneficial influence is strikingly apparent. Here, as in typhoid fever, the pulse is the best guide to its use. From three to six ounces of brandy, or a larger amount of port wine, may be given in the twenty-four hours—in desperate cases eight or even twelve ounces of brandy. Whatever the kind and amount of stimulant employed it is given in divided doses, well diluted, every two or three hours. For medicine a mixture containing carbonate of ammonia is generally prescribed in some such combination as the following: ℞ Ammonii carb., i.; tinct. calumbæ, ʒvj.; sp. chlorof., ʒi.ss.; aq. menth. pip. ad. ℥vj. M. sig: One tablespoonful in a wineglassful of water every three (or four) hours. A purgative may be needed at the outset; a colocynth and henbane pill, or, if the pulse is strong and the tongue coated, a mercurial—blue pill or calomel—may be given.

As has been already pointed out, the fever never calls for active remedies to reduce it, the employment of simple sponging, and sometimes of liquor ammoniæ acetatis being all that is needed. Antipyretics, such as antipyrin and antifibrin, are never given. If the patient passes into an adynamic state, of course the alcohol is given in greater amount, sometimes in the form of champagne. In this condition great benefit seems to follow the use of quinine, which may be given in pill, in 2-grain doses, three, four, or six times a day. Digitalis is also sometimes ordered. Should it be necessary to procure sleep, 10 or 15 grains of sulphonal are commonly administered in the evening.

The line of treatment here described is in the main that which is advocated by Dr. A. P. H. Waters, and was carried out by him for many years, first in the Northern Hospital, and afterwards in the Royal Infirmary. His results are given in his last work, "Contributions to Clinical and Practical Medicine," together with a brief summary of the main outlines of each case. Out of a total of 151 cases tabulated there were 11 deaths, a mortality of 7·28 per cent.

